# Serum proteomic profiles in CKCS with Mitral valve disease

**DOI:** 10.1186/s12917-017-0951-5

**Published:** 2017-02-07

**Authors:** Chiara Locatelli, Cristian Piras, Giulia Riscazzi, Isabella Alloggio, Ilaria Spalla, Alessio Soggiu, Viviana Greco, Luigi Bonizzi, Paola Roncada, Paola G. Brambilla

**Affiliations:** 1 DIMEVET, Department of Veterinary Medicine, University of Milan, Milan, Italy; 2 Fondazione Santa Lucia –IRCCS, Rome, Italy; 3 Istituto Sperimentale Italiano L. Spallanzani, Milan, Italy

**Keywords:** Proteomic analysis, Mitral valve disease, Dog, Cavalier King Charles Spaniel

## Abstract

**Background:**

Myxomatous mitral valve disease (MVD) is the most common acquired heart disease in dogs, and the Cavalier King Charles Spaniel (CKCS) is the most studied breed because of the high prevalence, early onset and hereditary component evidenced in the breed. MVD has different severity levels, and there are many practical limitations in identifying its asymptomatic stages. Proteomic techniques are valuable for studying the proteins and peptides involved in cardiovascular diseases, including the period prior to the clinical onset of the disease. The aim of this study was to identify the serum proteins that were differentially expressed in healthy CKCS and those affected by MVD in mild to severe stages. Proteomics analysis was performed using two-dimensional gel electrophoresis separation and a bioinformatics analysis for the detection of differentially expressed spots. In a comparative analysis, protein spots with a *p* < 0.05 (ANOVA) were considered statistically significant and were excised from the gels for analysis by MALDI–TOF–MS for protein identification.

**Results:**

Eight proteins resulted differentially expressed among the groups and significantly related to the progression of the disease.

In mild affected group versus healthy dogs complement factor H isoform 2, inhibitor of carbonic anhydrase, hemopexin, dystrobrevin beta isoform X7 and CD5 molecule-like resulted to be down-regulated, whereas fibronectin type-III domain-containing protein 3A isoform X4 was up-regulated.

In severe affected dogs versus healthy group complement factor H isoform 2, calpain-3 isoform *X*2, dystrobrevin beta isoform X7, CD5 molecule-like and l-2-hydroxyglutarate dehydrogenase resulted to be down-regulated.

Complement factor H isoform 2, calpain-3 isoform *X*2, dystrobrevin beta isoform X7, CD5 molecule-like and hydroxyglutarate dehydrogenase were found to be down-regulated in mild affected group versus healthy dogs. All of these proteins except complement factor H followed a decreasing trend according to the progression of the pathology.

**Conclusion:**

The differential expression of serum proteins demonstrates the possibility these might be valuable for the detection and monitoring of the disease. Further longitudinal studies are required to determine whether differential protein expression occurs sufficiently early in the progression of the disease and with sufficient predictive value to allow proteomics analysis to be used as an early detection and on-line diagnostic tool.

## Background

Myxomatous mitral valve disease (MVD) is the most prevalent acquired heart disease in dogs, and is the most common cause of left heart failure, particularly in older and small breed dogs [1].

The Cavalier King Charles Spaniel (CKCS) is an exception, and the interesting in study MVD in this breed comes from the evidence that CKCS shows the youngest onset and the highest incidence, compared with other breeds [1–5].

The cause of canine MVD is currently unknown, although a genetic predisposition has been proven [2, 4, 6].

MVD has also been increasingly considered as the result of certain effector proteins released in response to mechanical or chemical stimuli through signalling pathways rather than only an age-related disease [6]. The signalling mechanisms at the core of MVD have been investigated by the use of new technologies, and advances in proteomic techniques have improved the tools available for studying cardiovascular diseases [7]. The proteome reflects all proteins and peptides that may be related to certain genes and allows a more detailed evaluation of disease status [8, 9]. In 2009, Lacerda et al. conducted the first proteomic study on canine mitral valve leaflet tissue obtained postmortem from healthy dogs and from dogs with early and end-stage MVD. More than 300 proteins were identified, and 117 of these that were over- or under-expressed, which could provide mechanistic clues into the pathogenesis of MVD [3, 10].

In 2013, Gao et al. investigated plasma protein expression in human patients affected by degenerative or rheumatic valvular disease and compared it with healthy subjects [8]. They reported that few proteomic studies on MVD have been done, and most of them studied tissue, whereas the serum or plasma are ideal sources for proteome analysis, as they are easily sampled and are able to analyse processes in specific anatomical compartments because blood flows in all tissues and is therefore able to take up proteins secreted or shed by tissues [8]. Authors used a similar experimental approach with the difference that they used the depletion of high abundant proteins and described that Carbonic anhydrase protein was over-represented.

In 2013, Tan et al. applied proteomic methods to plasma samples collected from humans with mitral regurgitation (MR) and with or without mitral valve prolapse (MVP) [11]. This study revealed four proteins differentially expressed with a presumptive role in the pathogenesis of MVP: haptoglobin, platelet basic protein and complement component C4b were down-regulated and fibronectin was up-regulated in MR patients with MVP versus the control cases (MR) [11]. In 2014, Xuan et al. applied his experience in plasma proteomic studies to identify plasma protein changes in congenital heart disease with regard to potential clinical significance [8, 9, 11].

At present, in veterinary medicine there is no treatment for the disease itself that can be applied on a large population of patients (i.e. valve replacement and repair surgery), and so the prevention of its occurrence is the only strategy to reduce the incidence in dogs.

To date in order to reduce the prevalence of MVD in CKCS, a breeding program has been conducted in Sweden since 2001. This program was based on heart auscultation, on pedigree analysis and on the indication that dogs under the age of four years should not be bred [12]. The effects of the breeding program were evaluated by comparing the prevalence of heart murmur in CKCS at 6 years of age in 2007 and 2009. No decrease in the prevalence of the murmur was detected, indicating that the breeding program did not achieve the desired effects [12, 13].

In 2016 the results of a mandatory breeding scheme based on cardiac auscultation and on echocardiographic detection of mitral valve prolapse, performed in Denmark from 2002 to 2011 were published. The study evidenced a significant reduction in risk of having a mitral regurgitation murmur caused by MVD after a 8 to 10 year period [13]. Nevertheless same authors reported of limitations to a wide application of the program, and that may affect the use of this method in breeding schemes including multicentre clinical examinations (differences in echocardiographic equipment, technical settings, replacing of the operators, and the changing in the overall observer performance) [13].

To our knowledge there has been extensive research into attempting to diagnose MVD, and more particularly, to diagnose the onset of heart failure in dogs, but none in which proteomic techniques on CKCD clinical patients have been used. To date, there are no reports of serum proteomic changes in dogs affected by MVD. The aim of this study was to evaluate the serum protein profile of healthy and MVD affected CKCS in order to evaluate the physiological changes and the modifications caused by MVD. These findings could provide new insights into our understanding of the underlying mechanisms and the changes from mild to severe stages. The differential expression of the serum proteins moreover could be helpful together with the current techniques in the evaluation of the stage of the pathology, of the drug administration outcome and of the disease progression.

## Methods

The study was conducted on privately owned CKCS prospectively recruited at the Cardiology Unit of the Department of Veterinary Medicine, University of Milan (DiMeVet).

A convenience sample of 12 CKCS was enrolled, 4 healthy dogs (control group) and 8 dogs affected by MVD.

All of the owners signed an informed consent before enrolling their dogs in the study. Each dog underwent a complete physical examination aimed at evaluating the general physical condition. The cardiovascular system was evaluated by checking for the presence/absence of a murmur, type and intensity of the murmur (grade I-VI/VI), if present, and the point of maximum intensity. Blood pressure was indirectly measured with a Doppler method according to the ACVIM consensus statement [14]. Once reliable consecutive readings were obtained, the mean of five consecutive blood pressure measurements was calculated [12, 15]. Blood was collected from the jugular vein into 5-mL serum tubes and 2.5-mL EDTA tubes after a 12-h fasting. A complete blood count (CBC) was performed for each dog, and serum samples were centrifuged within 30 min after collection at 3000 × g for 5 min, followed by aliquoting of the plasma. The total protein content and the blood glucose of each sample were immediately evaluated. Serum protein amount was determined using the Bradford dye-binding method (Protein Assay kit 2, Bio-Rad catalogue n° 5000002) according to the manufacturer’s instructions. Subsequently, serum biochemistry, including creatinine, urea and serum calcium, potassium, sodium and chloride levels, was analysed. The remaining serum was harvested and transferred into 1.5-mL plastic cryotubes, and the samples were stored at -80°C for subsequent analysis.

Thoracic radiographs in right lateral and dorsoventral recumbency were obtained for all of the dogs included. Each radiograph was evaluated for the presence/absence of cardiomegaly, left atrial/ventricle enlargement, venous congestion, and pulmonary edema.

Echocardiographic examination (2-D, M-mode, spectral, and colour-flow Doppler) was performed by two well-trained investigators using a machine equipped with a multi-frequency phased array probe (2.5–3.5 MHz)^1^.

The exam was performed according to a standard procedure with concurrent continuous electrocardiographic monitoring [16]. High-quality video clips were acquired and stored using the echo machine software for off-line measurements. All measurements of interest were repeated on 3 consecutive cardiac cycles, and the mean value was used in the statistical analysis [17].

B-mode and M-mode echocardiography were used to define valve morphology and structures, including the type of lesions and the presence/absence of valvular prolapse, while the degree of mitral regurgitation (MR), if present, was assessed by the use of colour Doppler, calculating the maximal ratio of the regurgitant jet area signal to LA area (ARJ/LAA ratio) [17]. The left atrial to aortic root ratio (LA/Ao) was measured as previously described [4, 18]. Diagnosis of MVD was based on 2-D and colour Doppler echocardiographic findings: characteristic valvular lesions of the mitral valve apparatus and a demonstrated MR on the colour Doppler echocardiogram were considered as the definitive diagnostic criteria, as previously described [17].

The M-mode-derived end-diastolic volume index (EDVI) and the end-systolic volume index (ESVI) were calculated for each patient using the Teichholtz method [19]. The area length method was used for the calculation of 2-D derived parameters: EF%, EDVI and ESVI for each patient.

Based on body weight, the values for LVIDad (left ventricle internal diameter allometric diastole) and LVIDas (left ventricle internal diameter allometric systole) were also calculated according the formulas proposed by Cornell et al. (2004) [20].

The 12 enrolled subjects were stratified into three groups according to the guidelines for the diagnosis and treatment of chronic valvular heart disease established by the American College of Veterinary Internal Medicine Consensus Statement (ACVIM) [21].

Group H included 4 healthy subjects (stage A of ACVIM) with a normal clinical history, examination, thoracic radiography, echocardiography, serum biochemistry, and complete blood count (CBC) [21].

Group M included 4 asymptomatic affected dogs (stages B1 and B2 of ACVIM) with a systolic heart murmur, echocardiographic evidence of MVD and no radiographic signs of venous congestion or pulmonary edema [21].

Group S included 4 symptomatic CKCS (stage C and D of ACVIM) with clinical and radiographic signs of heart failure and echocardiographic evidence of MVD [21]. All of the dogs of the S group were treated with chronic pharmacologic therapy for heart failure including furosemide, angiotensin-converting enzyme inhibitors and pimobendan at the recommended dosage (Atkins et al. 2009). None of them received spironolactone or other potassium-sparing diuretics.

### Serological proteome analysis (SERPA)

Before analysis, the frozen serum was thawed slowly at room temperature [22, 23]. The serum samples were analysed through 2D electrophoresis. The first-dimensional analysis was performed using Immobiline Dry strips (pH 3-7 NL, length 7 cm, GE-Healthcare). Immobiline Dry strips were rehydrated overnight in a buffer containing 7 M urea, 2 M thiourea, 4% 3-[(3-Cholamidopropyl) dimethylammonio]-1-propanesulfonate hydrate (CHAPS), 1% threo-1,4-Dimercapto-2,3-butanediol, DL-Dithiothreitol, Cleland’s reagent (DTT), 2% ampholine pH 3.5–10 and 0.002% bromophenol blue. One hundred μg of protein was loaded on each IPG strip by cup loading at the cathodic side. Isoelectric focusing was performed using the Protean IEF (Isoelectric Focusing) Cell System (Bio-Rad). For IEF, the following protocol was used: 100 V (4h), 250 V (2h), 5000 V (5h), 5000 V (50000 Volt-hrs).

After the first dimension run was completed, the strips were equilibrated with a solution containing 6 M urea, 20% glycerol, 2% SDS and 50 mM Tris–HCl (pH 8.8), with the addition of 1% w/v DTT in the first step and 2.5% w/v iodoacetamide in the second step. For the second dimension, the proteins were separated by SDS-PAGE on 10% polyacrylamide gels using the Mini Protean System (Biorad, USA). Molecular weight protein markers (Precision Plus - BioRad) were applied on one end of the IPG strips. In the second dimension, the gels were run at 7.5 mA/gel for 20 min and then at 15 mA/gel until the bromophenol blue ran off of the gel. The gels were stained with Coomassie G250.

All images were acquired using an Image Scanner III (GE Healthcare, Uppsala). The gel images were imported into Progenesis SameSpot v4.5 software (Nonlinear Dynamics, UK) for analysis. All of the imported images were processed with Progenesis SameSpots to check image quality (saturation and dimension). The aligned images were then automatically analysed using the 2D analysis module for spot detection, background subtraction, normalization, and spot matching, and all spots were manually reviewed and validated to ensure proper detection and matching.

### Statistical analysis

The statistical analysis of the echocardiographic variables, the electrolyte concentrations and the haematological parameters was performed using SAS statistical software (SAS version 11 SW; SAS Institute, Cary, North Carolina, USA). Medians and ranges were calculated, and *p*-values less than 0.05 were considered statistically significant. The differences among the categories were assessed using the Kruskal-Wallis test, followed by a paired Wilcoxon test.

Statistical analysis of the proteomic data was performed using the Progenesis Stats module on the log-normalized volumes for all spots. The Stats module automatically performed a one-way ANOVA on each spot to evaluate the *p*-value between different groups, with *p*-values under 0.05 considered statistically significant.

### MALDI TOF MS protein identification

Single spots were excised from the Coomassie-stained 2DE gels, and the spots, previously reduced and alkylated, were digested with a solution of 0.01 μg/μl of porcine trypsin at 37 °C for 16 h. The reaction was stopped with 1% TFA in H_2_O (v/v). The peptides were desalted and concentrated by a C18 ZipTip (Millipore) and then co-crystallized with a solution of 0.5 mg/ml α-ciano-4-hydroxycinnamic acid (HCCA) on a Ground Steel plate (Bruker-Daltonics, Bremen, Germany) previously spotted with a thin layer of a 10 mg/ml HCCA solution. Mass spectra were acquired with an Ultraflex III MALDI-TOF/TOF spectrometer (Bruker-Daltonics) in a positive reflectron mode and processed with FlexAnalysis 3.1 software (Bruker-Daltonics) for the selection of the monoisotopic peptide masses. External and internal calibrations were performed. After exclusion of contaminant ions (known matrix and human keratin peaks), the created peak lists were analysed by the MASCOT v.2.4.1 algorithm (www.matrixscience.com) against the NCBI_201410 database restricted to Canis Lupus Familiaris (class: Other Mammalia). The query for database searching was performed with the carbamidomethylation of cysteines as a fixed modification, the oxidation of methionines as a variable modification, and one missed cleavage site allowed for trypsin at 70 ppm as a maximal tolerance. Mascot protein scores greater than 74 were considered significant (*p* < 0.05) for protein identification assignment. To confirm PMF identifications, the instrument was switched to LIFT mode; MS/MS spectra were acquired and processed by Flex-Analysis 3.0 software (Bruker-Daltonics).

## Results

The 4 dogs in the H group were significantly younger than those in the M group (2.3 vs 6.1 years, *p* = 0.0380) and those in the S group (9 versus 2.3 years, *p* = 0.0284). There were no significant differences by sex and weight between the three groups (Table 1). The normal reference values, the medians and ranges of the echocardiographic variables for all of the dogs included and for the different groups are reported in Table 2 [20, 24, 25].

**Table 1:** Population characteristics

ID	Age (years)	Sex	Weight (kg)	MVD severity
1	3	F	11,5	H
2	2	F	6,8	H
3	1,5	F	9	H
4	3	F	7	H
5	2	M	7,5	M
6	5,5	F	5	M
7	10	FS	9	M
8	7	F	9	M
9	9	M	8	S
10	7	M	12	S
11	11	FS	9,5	S
12	9	M	8	S

*MVD* mitral valve disease, *F* female, *M* male, *FS* neutered female, *H* healthy dogs, *M* mild affected, *S* severe affected

**Table 2:** Echocardiographic variables in all dogs and in each groups of severity

Variables M mode	Normal value	Over all population (12 dogs)	Group H (4 dogs)	Group M (4 dogs)	Group S (4 dogs)
LVIDs (mm)	* 15.2 ± 4.8	19.8 (15.9-31.4)	19.1 (16.2-20.7)	19.2 (17.7-22.6)	19.45 (15.9-31.4)
LVIDd (mm)	* 26 ± 4.7	30.95 (25.1-49.8)	29.75 (25.1-31.1)	30.1 (25.3-33.9)	39.65 (34.6-49.8)
LVIDas	0.71-1.26	0.94 (0.82-1.63)	0.92 (0.87-1.12)	1.11 (0.93-1.15)	0.92 (0.82-1.63)
LVIDasd	1.27-1.85	1.77 (1.22-2.7)	1.61 (1.22-1.77)	1.65 (1.30-1.91)	1.97 (1.86-2.7)
ESVI (ml/m^2^)	<30	31.9 (17-96)	23.9 (19.9-37.1)	37.9 (39.3-23.8)	36.5 (17.4-96.1)
EDVI (ml/m^2^)	<100	105.8 (43-287)	84.9 (43.4-104.7)	91.3 (59-125.7)	157.5 (120-287.9)
EF%	* 70 ± 9.20	67.5 (38-86)	69 (38-78)	61.5 (51-69)	83 (66-86)
Variables 2D					
LA/Ao	* 1.22 ± 0.31	1.3 (0.9-3.1)	1.2 (0.9-1.3)	1.2 (0.9-1.6)	2.14 (1.6-3.1)
ESVI (ml/m^2^)	<30	24 (18-42)	24.5 (18-30)	20 (17-26)	41 (40-42)
EDVI (ml/m^2^)	<70	53.5 (31-167)	45 (37-54)	54.5 (31-68)	123.5 (80-167)

LVIDd was significantly increased in dogs in group S compared with dogs in group H (*p* = 0.0304) and with dogs in group M (*p* = 0.0304). LVIDad was increased in group S compared with group H (*p* = 0.0304). M-mode-derived EDVI was significantly increased in dogs in group S compared with dogs in group H (*p* = 0.0304). The ratio of left atrial to aorta root was significantly increased in dogs in group S than in group M (*p* = 0.0304) and group H (*p* = 0.0304). No statistically significant difference was found in the LVIDs, LVIDas, ESVI (M-mode), ESVI and EDVI (2-D) FS% and EF% (M-mode and 2-D) among the groups

Data are reported as median and ranges (minimum-maximum). *Data are expressed as means values ± standard deviation

The electrolyte normal values and their levels in the H, M and S groups are reported in Table 3 [26]. Serum sodium levels showed a statistically significant decrease in subjects in the S group compared with the M and the H groups (*p* = 0.0228 and *p* = 0.0256, respectively), and serum calcium levels also decreased significantly in subjects belonging to the S group compared to the H group (*p* = 0.0304).

**Table 3:** Electrolytes levels, red blood cells count (RBCC) and hematocrit reported as median and ranges (minimum–maximum) in all dogs and in each groups of severity

	Normal Value	Over all population	Group H (4 dogs)	Group M (4 dogs)	Group S (4 dogs)
Serum Calcium (mg/dl)	8.98-11.82	10.6 (9-12.2)	11.65 (11.3-11.9)	10.6 (9.3-12.2)	9.75 (9-10.1)
Serum Potassium (mmol/l)	3.7 – 5.8	4.6 (4.3-5.2)	4.75 (4.5-4.9)	4.65 (4.3-5.2)	4.45 (4.3-4.6)
RBCC (x10^6^/ μl)	5.5-8.5	6.23 (5.64-7.08)	6.45 (6.16-6.87)	6.1 (5.64-7.08)	6 (5.45-6.28)
Hematocrit (%)	35 - 55	37.4 (32.2-40.1)	36.55 (33.8-40.1)	37.6 (37.4-38.4)	35.9 (32.2-40.1)

Serum potassium and chloride and the levels of other biochemical analytes (creatinine and urea) were not significantly different among the three groups.

Red blood cells and haematocrit values decreased in group S versus the M and H groups, but the differences were not statistically significant (Table 3) [27].

### Proteomics analysis (SERPA 2DE and MALDI-TOF-MS)

**Fig. 1:**
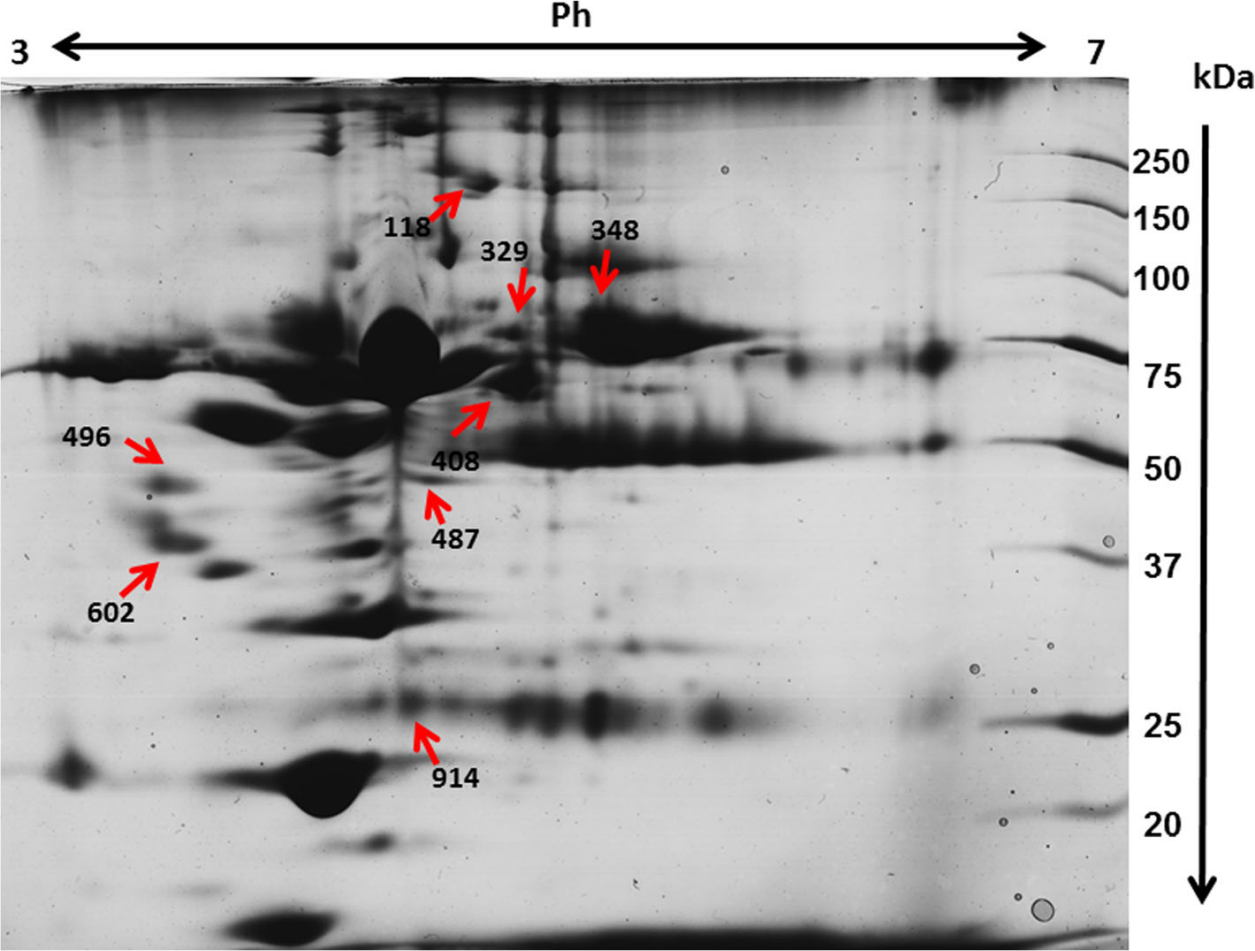
2D map showing the position of the protein spots analyzed by MALDI TOF MS. The red arrows and the numbers indicated correspond to the dataset showed in Table 3 indicating the differential expression pattern among experimental groups

Proteomics analysis highlighted a total of eight proteins differentially expressed among the groups (data shown in Table 4). Figure 1 shows the relative 2D map with differentially expressed proteins excised from the gel for MALDI- TOF MS analysis.

**Table 4:**
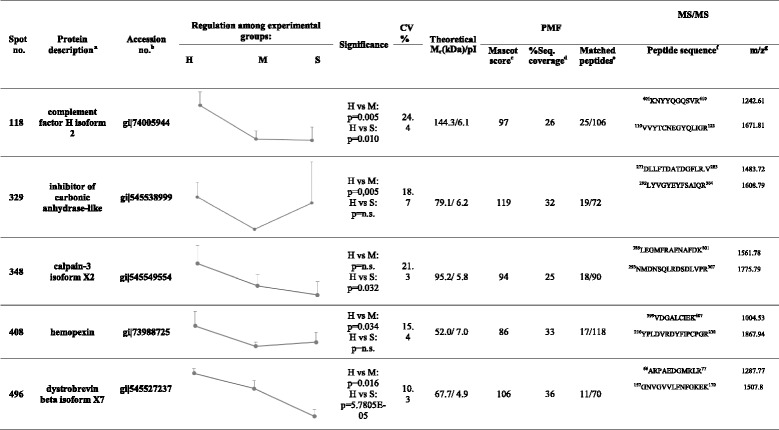
Protein identification by MALDI-TOF-TOF analysis

*H* healthy, *M* medium, *S* severe, *CV* variation coefficient, *Mr* relative Mass, *pI* isolectric point, ^a^Name of identified proteins; ^b^Accession No. according to database ^c^Peptide Mass Fingerprinting score calculated by MASCOT 2.4.1 algorithm (http://www.matrixscience.com) after database search; ^d^ sequence coverage; ^*e*^ Number of experimental peptides matched versus searched peptides; ^f^Aminoacidic sequence of the peptides identified by MS/MS analysis; ^g^Monoisotopic masses of the parent iones used for MS/MS analysis

Five proteins were found to be down-regulated and one protein was up-regulated (p ≤ 0.05) in group M versus H. The down-regulated proteins included complement factor H isoform 2 (gi|74005944), inhibitor of carbonic anhydrase (gi|545538999), haemopexin (gi|73988725), dystrobrevin beta isoform X7 (gi|545527237) and CD5 molecule-like (gi|545505255). Fibronectin type-III domain-containing protein 3A isoform X4 (gi|545537390) was found to be up-regulated in the M group versus the H group.

Five proteins were found to be down-regulated in group S versus group H: complement factor H isoform 2 (gi|74005944), calpain-3 isoform *X*2 (gi|545549554), dystrobrevin beta isoform X7 (gi|545527237), CD5 molecule-like (gi|545505255) and l-2-hydroxyglutarate dehydrogenase (mitochondrial isoform 2, gi|345804340). As shown in Table 4, complement factor H isoform 2, calpain-3 isoform *X*2, dystrobrevin beta isoform X7, CD5 molecule-like and hydroxyglutarate dehydrogenase were found to be down-regulated in group M versus group H. All of these proteins except complement factor H followed a decreasing trend according to the progression of the pathology.

## Discussion

To our knowledge, this is the first proteomic study on serum proteins in CKCS affected by MVD. In this study, the differential proteomic profiles of CKCS dogs affected by MVD with different degrees of severity, as well as healthy controls, has been analysed.

This study was focused mostly on proteins differentially expressed in mildly affected dogs (group M) in comparison to healthy controls in order to provide complementary tools for the early detection of this pathology and/or in the evaluation of its stage/severity.

The group M (or the mildly affected dogs) represented a valuable pool of samples for the study of this pathology in its subclinical status. Nevertheless, all groups have been analysed and, the proteomics profiling of advanced stages provided a more complete overview of the trends of the expression of each protein. The average expression of each protein in all groups of CKCS has been represented in Table 4.

Among all differentially expressed proteins, Hemopexin was found to be down-regulated in M group in comparison to H group. It is an acute-phase protein that can bind and transport haeme to the liver for breakdown and iron recovery, after which the free hemopexin returns to circulation [8]. Hemopexin also acts as a multifunctional agent in important processes such as iron homeostasis, antioxidant protection, and signalling pathways to promote cell survival and gene expression. This has important implications in several pathologic conditions not only associated with the release of heme but also in the control of the immune response and in the pathogenesis of renal and cardiovascular diseases [28]. The haemopexin down-regulation found in CKCS belonging to the M group is in accordance with the results reported by Ge Gao et al. [8]. In humans affected by degenerative mitral valve disease; however, these authors did not advance any hypothesis on the underlying mechanisms, and more studies were advocated [8, 29]. Moreover, Krekken et al. reported the relationship between hemopexin and angiotensin II through the regulation of the vascular angiotensin II type 1 receptor (AT1-R) [28]. These evidences could provide new hypotheses on the remodelling processes induced by this pathology especially in the light of their correlation with the disease. Finally, the decreased levels of the hemopexin we found in asymptomatic dogs could be related to the binding of the free heme produced by the haemolysis described in dogs affected by MVD due to the blood turbulences in the left atrium [30].

The carbonic anhydrase family (CAs) includes zinc metalloenzymes that catalyse the reversible hydration-dehydration of carbon dioxide and bicarbonate. CAs has a role in many physiological and biological processes including calcification, acid-base balance, ion transport, and bone absorption; in vitro, it also promotes the formation of CaCO_3_. Calcium salt precipitation is an important step in tissue calcification, and valvular calcification is one of the common and key pathological changes in MVD [8]. Using the same experimental approach, Gao and colleagues [8] identified the over-representation of Carbonic anhydrase 1 in human patients of Valvular Heart Diseases. This result seems to be consistent with the down-regulation of the inhibitor of carbonic anhydrase we found in mildly affected dogs. This result could be linked to the expression of calcium and to an improper mineralization process by the acceleration of calcium salt deposition on the mitral valve leaflets [8]. Moreover, the negative trend in the calcium levels in the serum from healthy dogs to those severely affected is in accordance with the down-regulation of the inhibitor of carbonic anhydrase evidenced in our study.

The fibronectin type-III domain-containing protein 3A isoform X4 was found to be over-represented in M vs H experimental group. This protein contains in its sequence domains of fibronectin that has been already described to be up-regulated [11] and its function is related to cell adhesion and is involved in the processes of tissue remodelling [11, 31].

In humans, the fibronectin type-III domain-containing protein 3A is as well involved in cell-cell adhesion among cells of the same organism according to GO annotations. Its putative role in this pathology is still unknown and needs further investigation but it can be considered important in the process of cardiac tissue remodelling.

In severely affected dogs, a significantly low sodium concentration was found in comparison with other groups; according to the literature, this could be due to the influence of the therapy, as Boswood observed that chloride tends to significantly decrease with the administration of treatment [32, 33]. The serum levels of potassium showed a decrease in the H group compared with the S group; however, this variation was not statistically significant.

All of the CKCS included in the study belonging to the S group received furosemide, which might be the reason for the lower potassium levels in the more severely affected dogs [32].

Furosemide could also be considered responsible for the significantly lower levels of serum calcium found in the dogs belonging to group S, as loop diuretics reduce the activity of the sodium-potassium-chloride cotransporter (NKCC2), which stimulates paracellular calcium reabsorption, causing an increase in renal calcium losses and hypocalcemia [34].

In our population, no significant alterations in haemoglobin, red blood cells or haematocrit were found.

Thrombocytopenia and macro thrombocytosis were also considered as a breed-specific idiopathic condition rather than a pathological feature in this study, consistent with other previously published studies [35–37]. Among other differentially expressed proteins, dystrobrevin beta isoform X7 was found to be down-regulated (Table 4) in the M and S groups in comparison to the H group, with a positive correlation with the disease severity. The role of dystrobrevin in hypertrophic cardiomyopathy has already been demonstrated in humans [38]. This evidence demonstrates how the impairment of the α-dystrobrevin gene (which also encodes for the β form) is linked to the development of cardiovascular diseases. Its strong down-regulation in MVD affected CKCS dogs, could suggest a role in the onset of this disease.

Complement factor H isoform 2 and CD5 molecule-like have also been found to be related to the severity of the pathology. The role of these proteins in the pathology still has to be elucidated; proteomic analysis of human plasma in chronic rheumatic mitral stenosis has revealed proteins involved in the complement and coagulation cascades [39]. However, their statistically significant down-regulation both in the M and S groups could suggest a role as molecular markers for this pathology.

This study reports a novel finding on canine MVD from the point of view of protein expression; however, there were several limitations. One of these concern the possibility that the changes in protein patterns, observed in each severity group, might be consistent with disease processes different from MVD. Nowadays the clinical examination and, above all the conventional Echo-Doppler exam represent the diagnostic gold standard of canine MVD. Nevertheless the lacking of post-mortem examination might represents a limitation to take in account [17]. Furthermore the relatively small sample size of each group could have resulted in less significant changes in the electrolyte concentrations compared with other studies, and, also the therapy could have influenced the electrolyte balance in the subjects belonging to the S group.

Another limitation was that the stratification of the patients takes into account the presence (group S) or absence (group M) of symptoms, and the ACVIM classes B1 and B2 CKCS are in the same strata. This limit must be considered in the next study, as the cardiac remodelling process that could be present in ACVIM class B2 and not in B1 could cause different protein expression patterns.

This is a pilot study and clearly further prospective cohort researches are needed to confirm our results, however, finding the same proteins on all groups of dog could be considered a confirmation of their functional importance, and encourage further investigations in studying the protein expression patterns in dogs affected by MVD.

## Conclusion

The genetic and the proteomics studies of MVD are important for providing additional information for the comprehension of this pathology and for the evaluation of the outcome of this disease. Obtained results and further studies on these different parameters may contribute to the improvement of the genetic selection of pure breeds to reduce the incidence of this pathology. Moreover, protein identified that were found to be positively or negatively related to the pathology may be helpful in the future for the diagnosis, for choosing the best therapeutic approach and for evaluating the therapeutic response to improve the quality of life and the clinical outcome in dogs affected by MVD.

## Abbreviations

ACVIM: American college of veterinary internal medicine
CKCS: Cavalier King Charles Spaniel
EDVI: End diastolic volume index
EF: Ejection fraction
ESVI: End systolic volume index
F: Female
FS: Neutered female
H: Healthy dogs
La/Ao: Left auricle to aorta ratio
LV: Left ventricle
LVIDad: Left ventricular internal dimension in diastole normalized for the body weight based on an allometric equation
LVIDas: Left ventricular internal dimension in systole normalized for the body weight based on an allometric equation
LVIDd: Left ventricular internal dimension in diastole
LVIDs: Left ventricular internal dimension in systole
M: Male
M: Mildly affected
MR: Mitral regurgitation
MVD: Mitral valve disease
S: Severely affected
